# Enterovirus A71-associated acute flaccid paralysis in a pediatric patient: a case report

**DOI:** 10.1186/s13256-023-04041-6

**Published:** 2023-07-19

**Authors:** Oluwafemi M. Akinnurun, Marco Narvaez Encalada, Julia Orth, Markus Petzold, Sindy Böttcher, Sabine Diedrich, Martin Smitka, Percy Schröttner

**Affiliations:** 1 Carl Gustav Carus Faculty of Medicine, Institute for Medical Microbiology and Virology, TU Dresden, Dresden, Germany; 2 Carl Gustav Carus Faculty of Medicine, Department of Paediatric Neurology, TU Dresden, Dresden, Germany; 3 National Reference Centre for Poliomyelitis and Enteroviruses, Robert Koch- Institute, Berlin, Germany; 4 Carl Gustav Carus Faculty of Medicine, Institute for Clinical Chemistry and Laboratory Medicine, TU Dresden, Dresden, Germany

**Keywords:** Enterovirus A71, Acute flaccid paralysis, Enterovirus PCR, Case report, Pediatric infectious diseases

## Abstract

**Background:**

Enterovirus A71 is one of the causative agents of hand, foot, and mouth disease, which is usually a self-limiting disease. Complications of enterovirus infection are also very rare. However, when such complications occur, they can lead to serious neurological diseases or even death.

**Case presentation:**

In this report, we describe a case of enterovirus A71-associated acute flaccid paralysis in a 13-month-old Caucasian girl that was managed in our hospital. The patient presented with sudden onset of left arm paresis that could not be attributed to any other cause. Establishing a diagnosis was furthermore complicated by negative virological investigations of cerebrospinal fluid and non-pathological radiological findings. A polymerase chain reaction test of the child’s stool sample however tested positive for enterovirus and sequencing results revealed the presence of enterovirus A71. A previous history of febrile gastroenteritis just before the paresis started also supported the suspected diagnosis of enterovirus-associated acute flaccid paralysis. Following this, the child was treated with intravenous immunoglobulin over 5 days and a remarkable improvement was observed in the child’s paresis.

**Conclusion:**

This case report describes a possible complication of enterovirus A71 infection in a child. It also highlights the prolonged detection of enterovirus in the child’s stool sample as compared with cerebrospinal fluid weeks after the primary infection occurred. Finally, it shows the need for increased clinical and diagnostic awareness especially in the management of sudden and unknown causes of paresis or paralysis in children.

## Background

Enterovirus A71 (EV-A71) is a member of the *Picornaviridae* family and is one of the main causative agents of hand, foot, and mouth disease (HFMD) [1–5] In addition to causing HFMD, EV-A71 is also capable of causing severe neurological diseases such as aseptic meningitis, brainstem and/or cerebellar encephalitis and acute flaccid paralysis (AFP) [1, 6]. The virus was first isolated in 1969 and described by Schmidt in 1974 [7]. Since then, EV-A71 infections have been reported worldwide with large epidemics occurring in the Asia–Pacific region and Eastern Europe [1, 2, 4, 8]. According to Zhu *et al.*, for example, 10,717,283 cases of EV-A71 infection were registered in China between 2008 and 2014, of which 3046 were fatal [9]. In addition, Akhmadishina *et al.* reported an outbreak in a childcare facility in Russia. A total of 78 children were affected and one child died from the disease [10]. Increased detection of EV-A71 strains has also been reported in Germany recently [11, 12]. However, there is a paucity of detailed clinical data from these cases as these are not being reported [12].

The majority of enterovirus infections usually have a self-limiting course or resolve spontaneously without complications [1, 2, 13, 14]. Advances in clinical diagnostic methods and treatment options have also helped to improve the management of these infections. However, complications of enterovirus infections such as AFP remain clinically relevant because they are associated with persistent to significant neurological deficits that persist long after the initial infection occurs [15–17]. While several clinical reports on the association of AFP with other members of the enterovirus family do exist, there are only very few clinical descriptions of EV-A71-associated AFP. In this report, we describe a case of EV-A71-associated acute flaccid paralysis in a 13-month-old girl that was managed in our hospital, thereby providing a clinical description of this disease complication.

## Case presentation

### Case history

A previously healthy 13-month-old Caucasian girl, accompanied by her mother, presented to the emergency department of a local hospital in Saxony, Germany due to sudden onset of left arm weakness that started on the same day of presentation. One week prior to this, the child had a febrile gastroenteritis that subsided after 4 days. The left arm weakness however persisted in the local hospital, leading to a referral to the pediatric orthopedic department of our tertiary institution 2 days later. After clinical examination, the child was discharged with the recommendation of close observation and control.

Following no improvement of the left arm paresis, the child was readmitted to the pediatric orthopedic unit. Initially, there was a suspicion of a pulled elbow, but this was later excluded as no subluxation or dislocation could be seen on magnetic resonance tomography of the shoulder and arm. There was also no further diarrhea or vomiting since the initial gastroenteritis. The mother however reported a slight reduction in fluids intake by the child, albeit with normal feeding. There were also no abnormalities in the child’s urination. The child’s development had been regular as expected with no prior medical conditions nor history of regular medication use by the child. The child lives with her parents and goes regularly to a creche.

### Examination findings

The general, cardiovascular, respiratory, and abdominal examinations were all non-significant. The initial neurologic examination revealed a left arm paresis with the left arm lying in a pronation position. Pressure-pain could be elicited in the left shoulder and handgrip was present. The biceps and triceps tendon reflexes were reduced in the left arm as compared with the right arm. All other neurologic findings were non-significant.

### Diagnostic workup

Native and contrast medium-enhanced magnetic resonance tomography (MRT) of the skull and cervical vertebra were all without pathological findings and no malformations or demyelinations could be seen. Nerve conduction studies (nerve conduction velocity and somatosensory evoked potentials tests) revealed regular motor and sensory electroneurography and no signs of axonal or demyelinating neuropathy. Blood count results showed thrombocytosis (649 Gpt/l) and increased hemoglobin counts (8.4 mmol/l). All other hematological and biochemical parameters were non-significant. Cerebrospinal fluid (CSF) analysis revealed a disruption in the blood–brain barrier permeability with an increased CSF/serum albumin ratio (5.1 × 10^3^) and an increase in the number of diverse cells in CSF (+16%). All other CSF data were no-nsignificant.

Bacteriological and mycological CSF cultures were negative, serological analysis for *Borrelia* spp. and *Mycoplasma pneumoniae* in CSF as well as their respective antibody indices were negative. Viral polymerase chain reaction (PCR) test for herpes simplex virus type 1 and 2, enterovirus, and varicella zoster virus in CSF were all negative. Reverse-transcriptase PCR for enterovirus in stool was however positive.

The CSF sample was also reviewed by the pathology department of our hospital, where an assessment of lymphocytic inflammatory changes was made. Also there was no presence of tumor cells belonging to the non-Hodgkin’s lymphoma group or to leukemia. Considering the whole constellation with acute onset of left arm weakness in the child, history of febrile gastroenteritis, neurological examination findings of decreased deep tendon reflexes, CSF pleocytosis and positive PCR finding of enterovirus in the child’s stool sample, a suspected diagnosis of enterovirus-associated acute flaccid paralysis was made.

## Treatment and outcome

The findings and suspected diagnosis were discussed with the child’s parents, and after consent was obtained, an immunomodulatory therapy was initiated with intravenous immunoglobulin (0.4 g/kg/day) for 5 days under close monitoring and with regular physiotherapy. The therapy was well tolerated and there were no complications. To confirm the positive PCR result and to conduct assignment to an enterovirus type, the stool sample was sent to the German national reference centre for poliomyelitis and enteroviruses at the Robert Koch Institute (RKI), where EV-A71 (genotype C1) could be detected by RT-PCR and subsequent sequencing of the VP1 part of the genome [12].

After 2 days of immunoglobulin therapy, there was a remarkable improvement in the left arm paresis and the child was able to move her left arm to play, albeit with reduced elevation, anteversion and retroversion. Also, the biceps, radioperiosteal and triceps tendon reflexes were still reduced in the left arm as compared with the right arm. The enteric symptoms were also fully regredient and there were no other symptoms in the child.

**Fig. 1:**
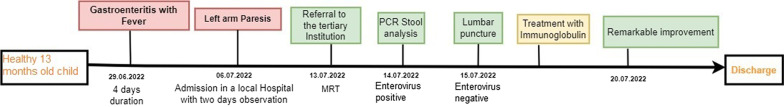
Timeline of events and clinical outcome

Following further improvement, the child was discharged after 7 days of admission (Fig. 1) in a stable and improved state. Neurological examination at discharge showed slight movement restrictions in the left arm, particularly proximal, with incomplete abduction, anteversion and retroversion against resistance. Also the biceps, radioperiosteal and triceps tendon reflexes were still slightly reduced in the left arm as compared with the right arm. Neurological rehabilitation was not desired by the child’s parents, however regular ambulant physiotherapy was recommended to help with the remaining paresis.

## Discussion and conclusion

EV-A71 infection usually manifests as HFMD, mainly in the South East Asia region, mostly with a self-limiting course or resolving spontaneously without complications [1, 2, 13, 14]. However, a small proportion of children with EV-A71 infection do develop complications [2]. Complications of EV-A71 infection such as AFP are of clinical relevance because these have been shown to be associated with persistent to significant neurological deficits, long after the initial infection occurs [15–17]. Data from EV-A71 epidemics and studies show that most of the hospitalized patients were children of 5 years of age or younger [4, 18], with a high case fatality rate in this age group [4]. The majority of patients with neurological complications of EV-A71 infection have also been under the age of 5 years [2], thus placing children 5 years of age or younger at particular risk of EV-A71 and its complications. This makes it even more crucial to raise awareness of the dangers of EV-A71 infection and its complications and to advocate for early identification and management of the disease. Our case patient clearly falls into this category of at-risk population for EV-A71 complications.

EV-A71 is predominantly transmitted via the fecal–oral route, for example, by contaminated hands either directly from vesicles on the hands, secretions from the respiratory tract, or from feces [2, 19, 20]. The transmission rates of EV-A71 among siblings has been estimated to be about 84% [8]. It has also been postulated that about 50% of asymptomatic, infected adults do often spread the disease to preschool children [2]. Thus, there are many possible routes by which our case patient could have come into contact with EV-A71. Contact tracing would be helpful here to help identify the likely infection source and possibly contain the disease from spreading further.

With the combination of acute onset of left arm weakness in the child, history of febrile gastroenteritis, neurological examination findings of limb weakness and decreased deep tendon reflexes, CSF pleocytosis, and positive PCR finding of enterovirus in the child’s stool sample, our case fulfilled six of the seven criteria proposed by Murphy *et al.* for the diagnosis of acute flaccid myelitis (AFM) [21]. However, the authors noted that MRT abnormalities could be subtle early in the disease and even be interpreted as normal in the clinical setting [21]. Helfferich *et al.* also described a case of EV-D68-associated AFM in a child that did not show MRT abnormalities even upon repeated scans [22]. In our case, the patient had acute flaccid paralysis of her left arm that was not accompanied by any abnormalities in the MRT of the skull or cervical vertebra or in the nerve conduction studies. Other possible causes of bacterial, viral, or fungal encephalitis were also excluded. The only hint to the possible etiology of AFP in our case patient was from the PCR of the stool sample that tested positive for enterovirus. These findings led to our suspected diagnosis of EV-A71-associated acute flaccid paralysis. EV-A71 was however not detected in the CSF sample. The negative test result of the CSF sample could however be due to the long period between acute infection and CSF sample collection (Fig. 1).

Generally, stool samples as well as cerebrospinal fluid and clinical samples from the throat, swabs from ulcers/vesicular fluid, and serum samples are suitable for establishing the diagnosis of EV-A71 infection [13, 18, 20]. However, samples taken from sterile body regions (for example, CSF) do provide results with a significantly higher reliability for the detection of EV-A71 [13]. Harvala *et al.* reported that enterovirus viral loads in stool, blood, and respiratory samples are usually higher than in CSF samples [20]. Ooi *et al.* also reported that EV-A71 shedding from the gastrointestinal tract (via the throat or rectum or in stools) is usually prolonged whereas sterile sites such as CSF usually have very low viral load [23]. This low viral load of EV-A71 in CSF that has been reported could also explain the nondetection of EV-A71 in the CSF sample. According to Han *et al.*, EV-A71-specific sequences can be detected from throat swabs for up to 4 weeks, however with continuously decreasing sensitivity, while detection from stool samples is possible for up to 5 weeks [24]. Our report also supports the prolonged detection of enterovirus in stool sample.

Enteroviruses can also be detected by cell culture methods. This method is however work and time intensive. For this reason, viral culture and isolate typing is primarily conducted in specialized laboratories. There is currently no specific antiviral therapy for EV-A71-related diseases [18]. Intravenous immunoglobulin and milrinone therapy has however been associated with significantly improved patient survival and decreased mortality in cases of severe brainstem encephalitis [18].

Currently, three inactivated EV-A71 vaccines have been developed, with all three appearing to be effective in preventing hand, foot, and mouth disease, herpangina, or both in children (6 months to 6 years of age) for periods of 11–13 months after intramuscular administration of two doses of vaccine [8, 25, 26]. However, these vaccines are not licensed for use in the European Union. Infection control approaches such as hand-washing and reducing contacts with infected people can help to prevent the spread of the disease, however the efficacy of these approaches is dependent on the presence of clinical and laboratory surveillance of EV-A71 infections in the community to provide early warning of impending epidemics [1].

In Germany, enterovirus surveillance is conducted by the RKI on the basis of laboratory results and pseudonymized patient data [12]. The number of AFP cases investigated between 2010 and 2022 ranged from 29 to 76 per year, with an enterovirus positive rate between 6.5% and 14%. In total, 21 different enterovirus types have been detected in patients with AFP in Germany so far, with EV-A71 being the most frequently identified enterovirus type [27]. Although there was a general decline in enterovirus case detections in Germany during the severe acute respiratory syndrome coronavirus 2 (SARS-CoV-2) pandemic, this case report shows that enterovirus infections and their complications still occur and should be kept in mind by clinicians, virologists and infectious disease specialists when managing sudden and unknown causes of flaccid paralysis, particularly in children.

## Data Availability

The clinical data obtained from this case report are not publicly available due to data protection rights.

## Abbreviations

EV-A71: Enterovirus A71
HFMD: Hand, foot, and mouth disease
AFP: Acute flaccid paralysis
MRT: Magnetic resonance tomography
CSF: Cerebrospinal fluid
PCR: Polymerase chain reaction
RKI: Robert Koch Institute
